# Type of Continued Fever

**Published:** 1858-10

**Authors:** 


The type of continued fever.

Dr. Murchison issues a brochure *On the Changes which are supposed to have taken place in the Type of Continued Fever.* He maintains that so-called relapsing fever has been at all times remarkable for its small mortality as compared with that of ordinary typhus, and that in computing the influence of treatment by blood-letting, no inference can be correct which classes the relapsing fevers with the more ordinary forms of typhus and enteric fevers, and adduces therefrom the view that a change has taken place in the type of fevers generally. Having collated many facts, he offers in conclusion the following inferences from them:

"1. In comparing the mortality from continued fever at different times and places, or for the purpose of judging of the merits of different plans of treatment, it is essential to take into account the form of fever which has prevailed.

"2. The small mortality, and the frequency of sudden improvement in the symptoms, which were observed to follow venesection in the epidemic of 1817-20, and which have been attributed to that practice, were characteristics of the relapsing form of fever which then prevailed, and have been equally characteristics of it at all times, even when blood-letting has never been resorted to.

"Consequently, it is not a legitimate argument in favour of a change in the constitutional type of fever, to contrast the mortality after blood-letting in the *relapsing* epidemic of 1817-20, with what would be the effects of bleeding in the *typhus* of the present day."

